# A positive feedback between cholesterol synthesis and the pentose phosphate pathway rather than glycolysis promotes hepatocellular carcinoma

**DOI:** 10.1038/s41388-023-02757-9

**Published:** 2023-06-26

**Authors:** Junjie Hu, Ningning Liu, David Song, Clifford J. Steer, Guohua Zheng, Guisheng Song

**Affiliations:** 1 School of Pharmacy, Hubei University of Chinese Medicine, Wuhan, PR China; 2 Department of Medicine, University of Minnesota Medical School, Minneapolis, MN 55455 USA; 3 American High School, Fremont, CA USA; 4 Department of Genetics, Cell Biology and Development, University of Minnesota, Minneapolis, MN 55455 USA

**Keywords:** Cancer metabolism, Non-coding RNAs

## Abstract

Hepatic cholesterol accumulation and hypercholesterolemia are implicated in hepatocellular carcinoma (HCC). However, the therapeutic effects of cholesterol-lowering drugs on HCC are controversial, indicating that the relationship between cholesterol metabolism and HCC is more complex than anticipated. A positive feedback between cholesterol synthesis and the pentose phosphate pathway (PPP) rather than glycolysis was formed in tumors of c-Myc mice. Blocking the PPP prevented cholesterol synthesis and thereby HCC in c-Myc mice, while ablating glycolysis did not affect cholesterol synthesis and failed to prevent c-Myc-induced HCC. Unexpectedly, *HMGCR* (3-hydroxy-3-methylglutaryl-CoA reductase) and *G6PD* (glucose-6-phosphate dehydrogenase), the rate-limiting enzymes of cholesterol synthesis and the PPP, were identified as direct targets of microRNA-206. By targeting *Hmgcr* and *G6pd*, microRNA-206 disrupted the positive feedback and fully prevented HCC in c-Myc mice, while 100% of control mice died of HCC. Disrupting the interaction of microRNA-206 with *Hmgcr* and *G6pd* restored cholesterol synthesis, the PPP and HCC growth that was inhibited by miR-206. This study identified a previously undescribed positive feedback loop between cholesterol synthesis and the PPP, which drives HCC, while microRNA-206 prevents HCC by disrupting this loop. Cholesterol synthesis as a process rather than cholesterol itself is the major contributor of HCC.

## Introduction

HCC is a lethal malignancy without effective therapeutic approaches [1]. The incidence rate of HCC nearly matched its mortality, demonstrating the aggressiveness of this malignancy and limited therapeutic options [1, 2]. Although hepatitis B (HBV) and C (HCV) infection are considered the major causal factors of HCC, NAFLD/MAFLD (non-alcoholic fatty liver disease/metabolic associated fatty liver disease) is associated with an increasing incidence of HCC in the Western world [3, 4]. Given limited effects of chemotherapy and the insensitivity of HCC to radiotherapy, tumor extirpation represents the only choice for a long-term cure. Unfortunately, even with successful surgical removal, the presence of NAFLD/MAFLD is associated with an increased recurrence of tumor. Although immunotherapies have recently been approved to treat a variety of cancers, this approach is largely unsuccessful for the treatment of HCC. Further studies are needed to develop new drugs for this malignancy.

Cholesterol is an important component of cell membrane and required for cell growth; and the liver is the main organ for its synthesis. In addition, cholesterol plays an important role in modulating membrane trafficking and facilitating signal transduction [5]. An imbalance in cholesterol homeostasis can contribute to liver injury, which triggers HCC. However, the roles of cholesterol in regulating cancer development and the potential of therapeutically targeting cholesterol homeostasis is controversial [6]. It is reported that hepatic accumulation of cholesterol drives liver injury and subsequent HCC [7]. Hypercholesterolemia has been considered as a risk factor of HCC [8]. Statins, the drug for hypercholesterolemia, show the capacity to protect against the development and recurrence of HCC [9–11]. In contrast, other studies reported that statins failed to reduce the incidence of HCC in NAFLD-associated HCC patients [12]. In mice, atorvastatin exhibits no effect on N-nitrosodiethylamine-induced HCC [13]. The potential role of cholesterol-lowering drugs in treating HCC remains controversial. Cholesterol homeostasis requires collaboration between various organs, which ensures a balance between cholesterol absorption (in the intestine) and cholesterol synthesis and removal in the liver [14]. In addition to activation of cholesterol synthesis, enhancement of cholesterol absorption and impaired cholesterol removal also contributes to increased hepatic and serum cholesterol [14]. However, current studies described above only focused on hepatic and serum levels of cholesterol rather than cholesterol synthesis in HCC patients. In addition, statins function by driving hepatic uptake of cholesterol rather than cholesterol synthesis, which could potentially explain some of the controversy in the field. The pentose phosphate pathway (PPP) is a metabolic pathway parallel to glycolysis [15]. It is widely accepted that glycolysis is a major energy resource for cancer development, while the PPP produces NADPH (reduced nicotinamide adenine dinucleotide phosphate) and ribose 5-phosphate (R5P). R5P is a key substrate of DNA synthesis that is required for cell proliferation; and NADPH provides reducing power for cholesterol synthesis [15–17], suggesting that cholesterol synthesis is closely connected to the PPP and/or glycolysis. Our study was based on the notion that a positive feedback between cholesterol synthesis and the PPP promotes the development of HCC and cholesterol synthesis as a process rather than cholesterol is the major risk factor of HCC.

Amplification and overexpression of the *c-MYC* oncogene is frequently observed in HCC patients and is associated with increased aggressiveness and poor prognosis [18, 19]. In addition, c-Myc-induced HCC in rodents can recapitulate, in a highly reliable way, the phases of tumor initiation and progression that occur in humans [20]. Considering the role of cholesterol in HCC, we analyzed c-Myc mice and observed a significant increase in cholesterol and metabolites of the PPP in tumors. In addition, c-Myc also significantly impaired biogenesis of micoRNA-206 (miR-206) that directly targeted *HMGCR* (3-hydroxy-3-methylglutaryl-CoA reductase) and *G6DP* (glucose-6-phosphate dehydrogenase), the rate-limiting enzymes of cholesterol synthesis and the PPP. In this study, we tested the hypothesis that a positive feedback loop between cholesterol synthesis and the PPP promoted HCC development and the disruption of this loop by miR-206 prevented HCC development.

## Results

### Increased cholesterol synthesis in tumors of HCC patients

**Fig. 1:**
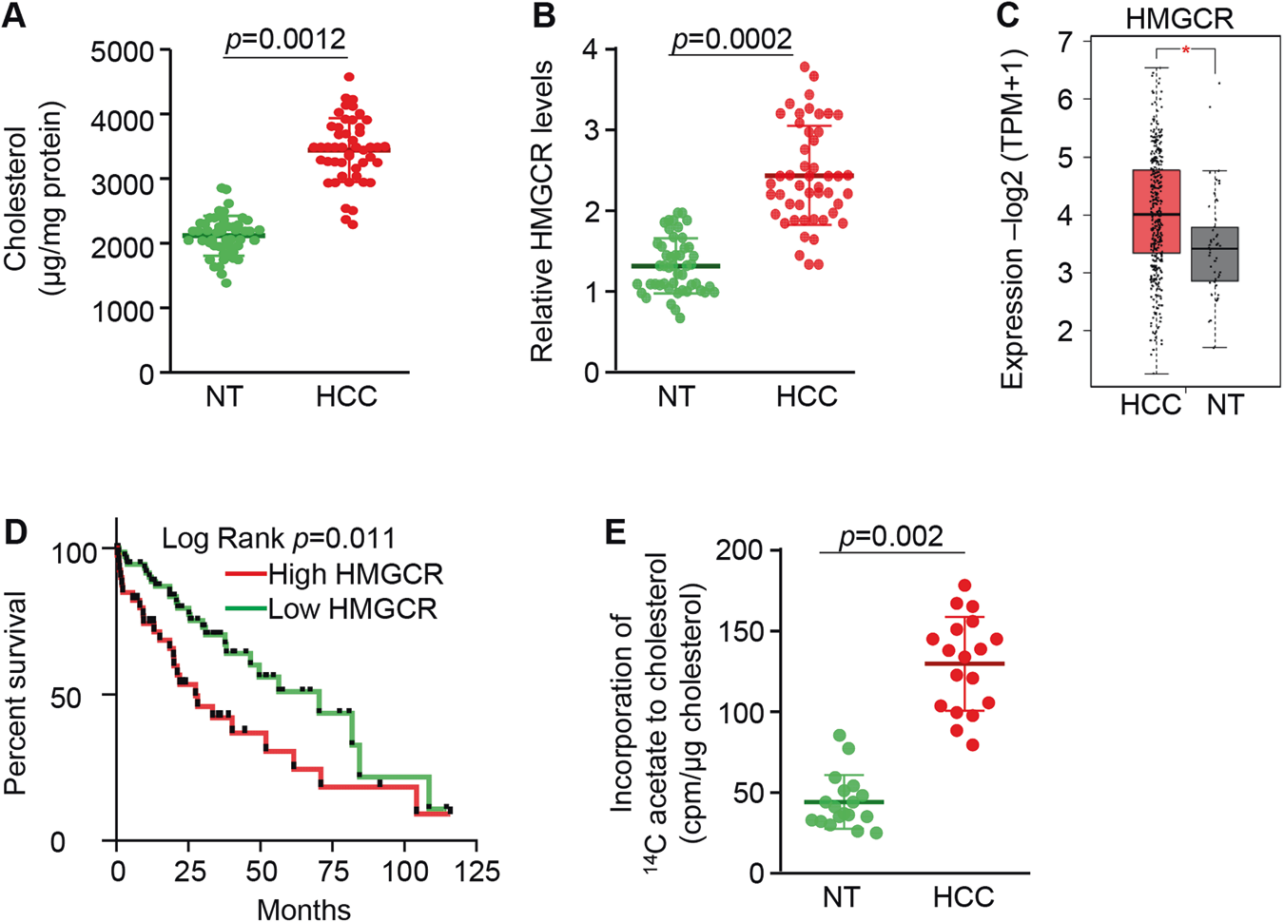
Activation of cholesterol synthesis predicted poor survival of HCC patients. **A** Levels of cholesterol in tumors and adjacent normal livers of HCC patients (*n* = 48). NT: adjacent normal liver tissues. **B** mRNA levels of *HMGCR* in tumors and adjacent normal livers of HCC patients (*n* = 48), as revealed by qRT-PCR. **C** A significant increase in *HMGCR* in tumors of HCC patients (*n* = 369) versus normal individuals (*n* = 50) from the TCGA database. NT: liver tissues from normal individuals. **D** High levels of *HMGCR* predicted poor survival of HCC patients in TCGA database. **E** Increased cholesterol synthesis in HCC tumors (*n* = 18) versus normal adjacent liver tissues (*n* = 18), which was reflected by increased incorporation of ^14^C acetate into cholesterol. Data represent mean ± SEM. *p < 0.05 (Fig. 1A–C, E: two-tailed Student’s *t* test; Fig. 1D: log-rank test).

Both cholesterol synthesis and excretion and absorption of cholesterol contribute to change in hepatic and blood cholesterol [14]. Hepatic cholesterol accumulation is implicated in HCC patients. However, the effect of cholesterol-lowering drugs on HCC is controversial. In HCC patients, compared to adjacent normal livers, cholesterol levels were significantly elevated in tumors (Fig. 1A). Hepatocytes are the major site of cholesterol synthesis. We next analyzed mRNA levels of *HMGCR*, the rate-limiting enzyme of cholesterol synthesis, in hepatocytes isolated from adjacent normal livers and HCC tumors. As expected, increased *HMGCR* mRNA was observed in malignant hepatocytes compared to normal hepatocytes (Fig. 1B). In TCGA database, compared to normal individuals (*n* = 50), HCC patients (*n* = 369) exhibited high expression of *HMGCR* (Fig. 1C), which was associated with poor survival (Fig. 1D). Increased hepatic cholesterol can be caused by activation of cholesterol synthesis, impaired cholesterol excretion and increased cholesterol absorption from the food [14]. Cholesterol-lowering drugs such as statins exhibit no effect on HCC in some types of HCC patients and mice [12, 13, 21, 22], leading us to speculate cholesterol synthesis as a process rather than cholesterol itself is the major contributor of HCC development. ^14^C-acetate incorporation into cholesterol was much greater in HCC tumors than in normal livers (Fig. 1E), suggesting activation of de novo cholesterol synthesis in HCC tumors. In sum, cholesterol synthesis was activated in tumors of HCC patients, and high levels of *HMGCR* in malignant hepatocytes are correlated with poor survival of HCC patients.

### c-Myc activated hepatic cholesterol synthesis, the pentose phosphate pathway and glycolysis in mice

**Fig. 2:**
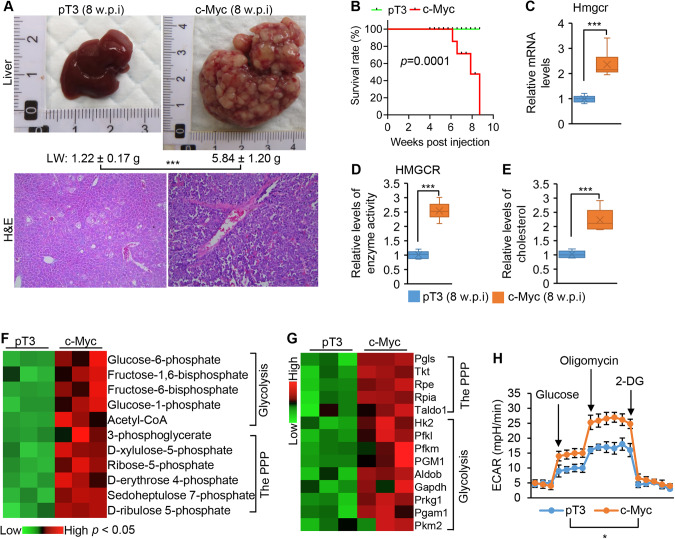
c-Myc drove cholesterol synthesis, the PPP and HCC development. **A** Representative photos of livers and H&E (10X) staining from FVB/NJ mice injected with pT3-EF1α (pT3, *n* = 6, 8 w.p.i) and pT3-EF1α-c-Myc (*n* = 6, 8 w.p.i). LW: liver weight. w.p.i: weeks post injection. **B** Kaplan–Meier survival curves of pT3 and c-Myc mice. **C**, **D** mRNA levels of *Hmgcr* and HMGCR enzyme activities in livers of pT3 and c-Myc mice. **E** Hepatic cholesterol levels in pT3 and c-Myc mice. **F** Levels of the metabolites of the PPP and glycolysis in livers of pT3 (*n* = 3) and c-Myc (*n* = 3) mice. **G** Upregulated genes controlling the PPP and glycolysis in livers of c-Myc mice compared to pT3 mice. **H** The extracellular acidification rate (ECAR) in hepatocytes isolated from pT3 and c-Myc mice after sequential additions of 10 mM glucose, 2 μg/mL oligomycin, and 100 mM 2-deoxyglucose. Data represent mean ± SEM. *p < 0.05 and *** *p* < 0.001 (Fig. 2A, C–H: two-tailed Student’s *t* test; Fig. 2B: log-rank test).

Almost 30% of HCC patients show *c-MYC* gene amplification or overexpression [23]. A positive correlation between *HMGCR* and *c-MYC* was observed in tumors of HCC patients from TCGA database (Supplementary Fig. 1A), indicating that c-MYC is a potential driver of cholesterol synthesis. HDI of c-Myc led to c-Myc accumulation and increased expression of *Hmgcr* in hepatocytes of mice (Supplementary Fig. 1B, C) and triggered development of HCC (Fig. 2A). However, no cholangiocarcinoma was observed in c-Myc mice (Supplementary Fig. 1D). All c-Myc mice died of HCC within eight weeks post injection of c-Myc, while 100% control mice were healthy at that time point (Fig. 2B). As we observed in HCC patients, mRNA levels of *Hmgcr*, enzyme activity of HMGCR, and hepatic cholesterol were significantly increased in tumors of c-Myc mice (Fig. 2C–E). Cholesterol synthesis, via acetyl-CoA, interfaces with de novo lipogenesis (DNL), glycolysis and the PPP [24], suggesting a possible mechanism of action. In fact, metabolites of glycolysis and the PPP were significantly increased in tumors of c-Myc mice (Fig. 2F); and acetyl-CoA, the major precursor of cholesterol synthesis, was increased in c-Myc mice (Fig. 2F). Consistent with an increase in the glycolytic and the PPP metabolites, expression of the genes controlling glycolysis and the PPP was significantly increased in malignant hepatocytes of c-Myc mice (Fig. 2G). An increase in the glycolytic rate was observed in malignant hepatocytes of c-Myc mice (Fig. 2H). In sum, c-Myc signaling promoted cholesterol synthesis, glycolysis and the PPP in hepatocytes.

### Ablation of the PPP reduced cholesterol synthesis and delayed growth of HCC, while ablation of glycolysis did not affect these processes in c-Myc mice

**Fig. 3:**
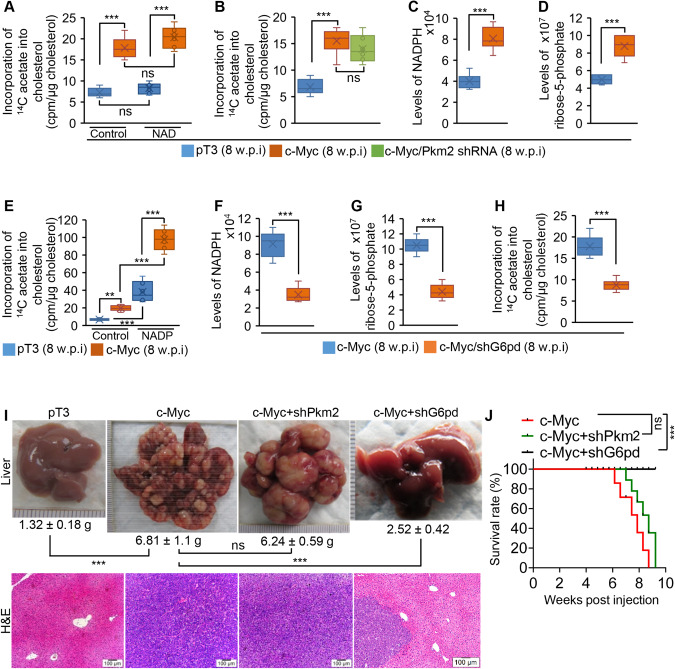
Ablation of the PPP reduced cholesterol synthesis and delayed growth of HCC, while ablation of glycolysis failed to affect these processes in c-Myc mice. **A** The rates of ^14^C acetate incorporation into cholesterol in livers of pT3 (*n* = 6) and c-Myc mice (*n* = 6) after treatment with diphosphopyridine nucleotide (NAD, a driver of glycolysis). **B** The rates of ^14^C acetate incorporation into cholesterol in livers of pT3 (*n* = 6), c-Myc (*n* = 6) or c-Myc and *Pkm2* shRNA (shPkm2, *n* = 6) mouse cohort. **C**, **D** Levels of NADPH and ribose-5-phosphate (R5P) in livers of pT3 and c-Myc mice. **E** The rates of ^14^C acetate incorporation into cholesterol in livers of pT3 and c-Myc mice after treatment with triphosphopyridine nucleotide (NADP, a promoter of the PPP). **F**, **G** Levels of NADPH and ribose-5-phosphate (R5P) in livers of c-Myc mice treated with scramble (*n* = 6) and *G6pd* shRNA (*n* = 6). **H** The rates of ^14^C acetate incorporation into cholesterol in livers of c-Myc mice injected with scramble (*n* = 6) and *G6pd* shRNA (*n* = 6). **I** Representative photos of livers and H&E (10X) staining from FVB/NJ mice injected with pT3-EF1α (pT3, *n* = 6, 8 w.p.i), c-Myc (*n* = 6, 8 w.p.i), c-Myc and *Pkm2* shRNA (c-Myc/shPkm2, *n* = 6, 8 w.p.i) or c-Myc and *G6pd* shNRA (c-Myc/shG6pd, *n* = 6). LW: liver weight. w.p.i: weeks post injection. **J** Kaplan–Meier survival curves of c-Myc, c-Myc/shPkm2 and c-Myc/shG6pd mouse cohorts. Data represent mean ± SEM. **p < 0.01; ***p < 0.001 and ns: no significance (Fig. 3A, B, E, I: two-way ANOVA test; Fig. 3J: log-rank test; and Fig. 3C, D, F–H: two-tailed Student’s *t* test).

c-Myc activated the PPP and glycolysis (Fig. 2F, G). Glycolysis produces pyruvate that can be converted to acetyl-CoA, a precursor of cholesterol synthesis. We next determined the effect of glycolysis on cholesterol synthesis. NAD is a driver of glycolysis [24, 25]. We, therefore, treated liver homogenates of pT3 and c-Myc mice with NAD. Although NAD enhanced glycolysis in both pT3 and c-Myc mice (Supplementary Fig. 2A), it did not affect cholesterol synthesis in both pT3 and c-Myc mice (Fig. 3A). We next deleted pyruvate kinase (PKM2), the rate-limiting enzyme of glycolysis in c-Myc mice, which ablated glycolysis (Supplementary Fig. 3A–C). Consistent with reduced glycolytic capacity, levels of pyruvate and acetate were significantly reduced in c-Myc mice with ablated *Pkm2* (Supplementary Fig. 3D, E). Unexpectedly, ablation of *Pkm2* at the time of c-Myc overexpression in murine livers did not affect cholesterol synthesis in c-Myc mice (Fig. 3B). However, cholesterol synthesis is still much higher in c-Myc mice compared to pT3 mice (Fig. 3A), indicating that other pathways activated by c-Myc such as the PPP might be able to drive cholesterol synthesis in mice.

The PPP is a metabolic pathway parallel to glycolysis, which shares a common starting molecule with glycolysis, glucose-6-phosphate (G6P). Two major products of the PPP are R6P and NADPH. As expected, NADPH and R5P as well as expression of *G6pd* was significantly increased in malignant hepatocytes from c-Myc mice (Fig. 3C, D, Supplementary Fig. 4A). NADPH, as a cofactor of HMGCR, is required for cholesterol synthesis. These established findings led us to speculate that the PPP is potentially involved in enhanced cholesterol synthesis in HCC. To test this speculation, we treated liver homogenates of pT3 and c-Myc mice with NADP. As expected, NADP treatment significantly increased enzyme activity of G6PD (Supplementary Fig. 4B), which in turn increased incorporation of ^14^C-acetate into cholesterol in liver homogenates from pT3 and c-Myc mice (Fig. 3E). Since the PPP is activated in c-Myc mice, cholesterol synthesis, as revealed by ^14^C-acetate labeling experiment, was much higher in c-Myc mice compared to pT3 mice (Fig. 3E). To confirm this speculation, we ablated the PPP via knocking down *G6pd* in hepatocytes of c-Myc mice (Supplementary Fig. 5). Knocking down *G6pd* significantly inhibited the PPP, which was reflected by a decrease in NADPH and R5P (Fig. 3F, G). Consistent with reduced NADPH that is required for cholesterol synthesis, incorporation of ^14^C-acetate into cholesterol was also significantly reduced in liver homogenates of c-Myc/shG6pd mice (Fig. 3H). Phenotypically, ablation of glycolysis did not affect growth of HCC, while knockdown of *G6pd* significantly delayed growth of HCC in c-Myc mice (Fig. 3I, J). In sum, the PPP at least in part is required for cholesterol synthesis and hepatocarcinogenesis in c-Myc mice.

### A positive feedback between the PPP and cholesterol synthesis drove DNA synthesis and cell proliferation

**Fig. 4:**
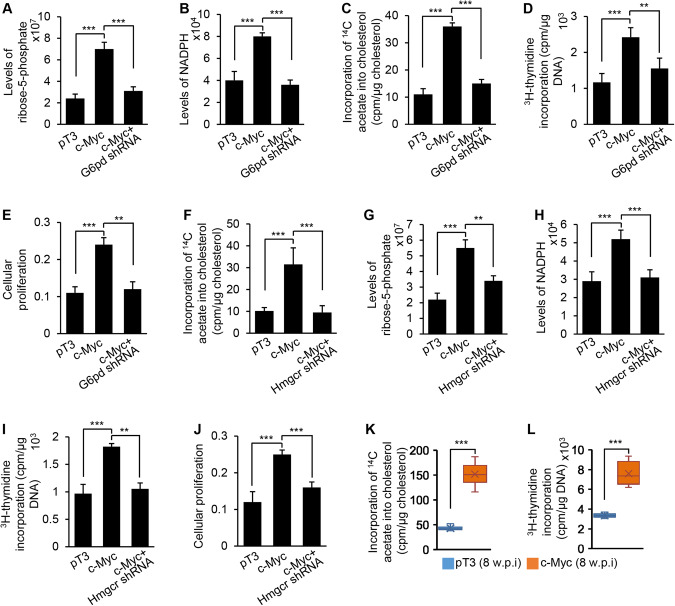
c-Myc promoted a positive feedback loop between cholesterol synthesis and the PPP, which drove DNA synthesis and cell proliferation. **A**–**D** Levels of R5P and NADPH, the rates of ^14^C-acetate incorporation into cholesterol, and the rates of ^3^H-thymidine incorporation into DNA in primary hepatocytes treated with pT3, c-Myc or a combination of c-Myc and *G6pd* shRNA. **E** Proliferation of hepatocytes transfected with pT3, c-Myc or a combination of c-Myc and *G6pd* shRNA, as revealed by MTT assay. **F**–**I** The rates of ^14^C-acetate incorporation into cholesterol, levels of R5P and NADPH, and the rates of ^3^H-thymidine incorporation into DNA in hepatocytes treated with pT3, c-Myc or a combination of c-Myc and *Hmgcr* shRNA. **J** Proliferation of hepatocytes transfected with pT3, c-Myc or a combination of c-Myc and *Hmgcr* shRNA, as per the MTT assay. **K**–**L** The rates of ^14^C-acetate incorporation into cholesterol and the rates of ^3^H-thymidine incorporation into DNA in livers of pT3 and c-Myc mice. Data represent mean ± SEM. **p < 0.01 and ***p < 0.001 (Fig. 4A–J: two-way ANOVA test; Fig. 4K, L: two-tailed Student’s *t* test).

Activation of the PPP produces more NADPH, which provides a cofactor for cholesterol synthesis. Enhancement of cholesterol synthesis should rapidly deplete NADPH, a major production of PPP. Therefore, we hypothesized that cholesterol synthesis and the PPP formed a positive feedback loop, which amplifies production of R5P, the substrate of DNA synthesis, and NADPH, co-factor for HMGCR. To determine if activation of the PPP drives cholesterol synthesis, DNA synthesis, and proliferation, three groups of hepatocytes were treated empty vector (pT3), c-Myc, or a combination of c-Myc and *G6pd* shRNA to knock down *G6pd* (Supplementary Fig. 6A). c-Myc overexpression enhanced the PPP, cholesterol synthesis, DNA synthesis and proliferation of hepatocytes (Fig. 4A–E), while knockdown of *G6pd* offset the effects of c-Myc overexpression (Fig. 4A–E). These findings indicated that activation of the PPP is required for c-Myc to drive cholesterol synthesis, DNA synthesis and hepatocyte proliferation. To test if enhancement of cholesterol synthesis promotes the PPP, DNA synthesis and proliferation, three groups of hepatocytes were treated with pT3 (control), c-Myc or a combination of c-Myc and *Hmgcr* shRNA (Supplementary Fig. 6B). c-Myc activated the PPP, cholesterol synthesis, DNA synthesis and hepatocyte proliferation; and knockdown of *Hmgcr* counteracted the effects of c-Myc (Fig. 4F–J). The significant increase in levels of ^14^C-acetate-labeled cholesterol and ^3^H-thymidine incorporation into DNA was observed in c-Myc mice (Fig. 4K, L). In sum, a positive feedback loop between cholesterol synthesis and the PPP enhanced production of cholesterol and R5P, which meets the needs for rapid growth and proliferation of malignant hepatocytes both in vitro and in vivo.

### MiR-206 repressed expression of *HMGCR* and *G6PD* in hepatocytes by binding to their 3’UTRs

**Fig. 5:**
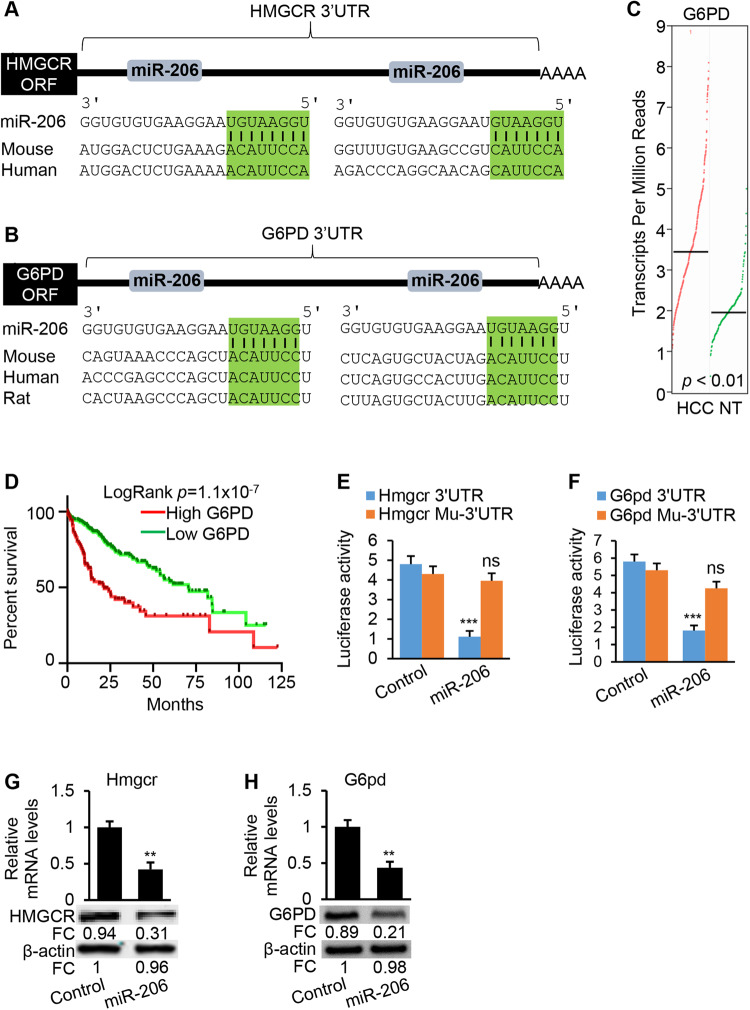
*HMGCR* and *G6PD* are the direct targets of miR-206. **A**, **B** Graphic representation of the conserved miR-206 binding sites within the 3’UTRs of *HMGCR* or *G6PD* between human and mouse. **C** Levels of *G6PD* in tumors of HCC patients (*n* = 369) and livers of normal individuals (*n* = 50) from the TCGA database. **D** High levels of *G6PD* predicted poor survival of HCC patients from the TCGA database. **E**, **F** miR-206 markedly reduced luciferase activity of the reporter construct containing wild-type 3’UTR of murine *Hmgcr* or *G6pd*. Mutation of two miR-206 sites within the 3’UTR of *Hmgcr* or *G6pd* nullified the ability of miR-206 to inhibit luciferase activity. **G**, **H** mRNA and protein levels of *Hmgcr* and *G6pd* in hepatocytes transfected with pT3-EF1α-miR-206-MM (control) or pT3-EF1α-miR-206. FC fold change. Data represent mean ± SEM. **p < 0.01, ***p < 0.001, and ns: no significance (Fig. 5C, E–H: two-tailed Student’s *t* test; Fig. 5D: log-rank test).

HMGCR and G6PD are the rate-limiting enzymes of cholesterol synthesis and the PPP. MicroRNAs (miRNAs) can simultaneously fine tune multiple pathways and exhibit the strong therapeutic potential for cancers and other diseases [26]. We next attempted to identify those miRNAs that can simultaneously target both *HMGCR* and *G6PD*. For this purpose, we analyzed murine and human Ago HITS-CLIP databases (high-throughput sequencing of RNAs isolated by crosslinking immunoprecipitation from Argonaute protein complex) of *HMGCR* and *G6PD*. Unexpectedly, miR-206 was identified as the only miRNA that can target human and mouse *HMGCR* and *G6PD* and 3’UTRs of mouse and human *HMGCR* and *G6PD* contains two miR-206 binding site [27, 28] (Supplementary Table 1). To exclude the false positive peaks of Ago-HITS-CLIP, we further used DIANA-microT-CDS to scan the 3’UTRs of murine and human *HMGCR* and *G6PD*, confirming the binding sites of miR-206 within the 3’UTRs of murine and human *HMGCR* and *G6PD*. 3’ UTRs of both human and mouse *HMGCR* and *G6PD* mRNAs are 100% complementary to the miR-206 5’ seed region, exhibiting the highest prediction scores and binding energy (Fig. 5A, B). In addition, levels of miR-206 were significantly reduced in malignant hepatocytes isolate from c-Myc mice (Supplementary Fig. 7). All these findings led us to focus on miR-206. In Fig. 1C, D, high levels of *HMGCR* predicted poor survival of HCC patients. Similarly, elevated levels of *G6PD* predicted poor survival of HCC patients in TCGA database (Fig. 5C, D). Inclusion of the 3’UTRs of *Hmgcr* or *G6pd* into the luciferase reporter constructs reduced luciferase activities upon co-transfection with miR-206 into Hepa1–6 cells (Fig. 5E, F). Mutation of the miR-206 binding sites within the 3’UTRs of *Hmgcr* and *G6pd* was necessary to completely offset the inhibitory effects of miR-206 on luciferase activities (Fig. 5E, F), suggesting that miR-206 directly recognized the predicted binding site within the 3’ UTR of *Hmgcr* and *G6pd*. miR-206 also reduced protein and mRNA levels of *Hmgcr* and *G6pd* in Hepa1–6 cells (Fig. 5G, H). MiR-206 was able to inhibit expression of *HMGCR* and *G6PD* in human hepatocytes by binding to their 3’UTRs (Supplementary Fig. 8A–C). In sum, *HMGCR* and *G6PD* are direct targets of miR-206 in both human and mouse hepatocytes.

### MiR-206 disrupted the positive feedback loop between cholesterol synthesis and the PPP by targeting *Hmgcr* and *G6pd*, which impaired DNA synthesis and proliferation of hepatocytes

**Fig. 6:**
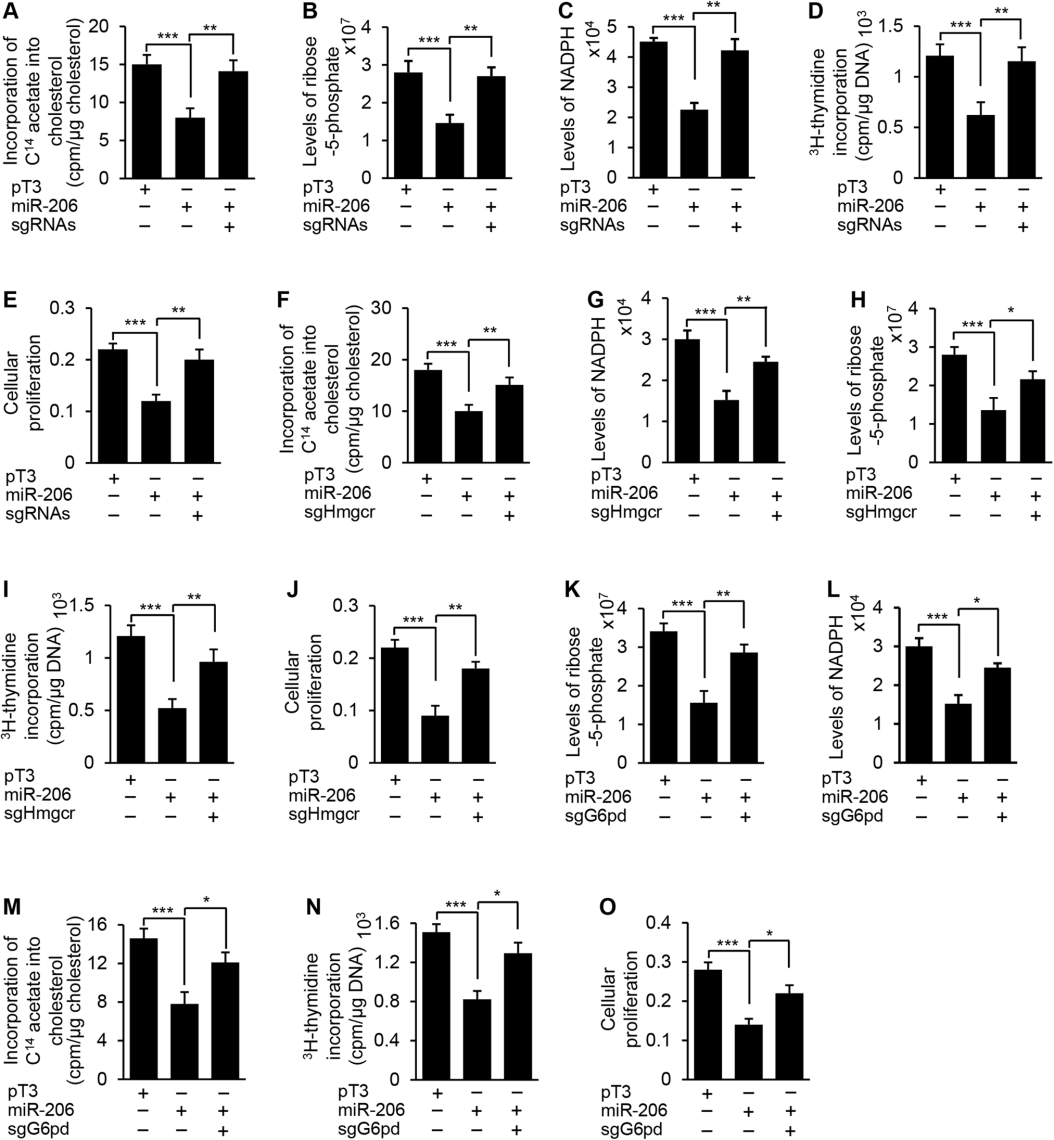
miR-206 inhibited DNA synthesis and cell proliferation by disrupting the positive feedback loop between cholesterol synthesis and the PPP. **A**–**E** The rates of ^14^C-acetate incorporation into cholesterol, levels of R5P and NADPH, the rates of ^3^H-thymidine incorporation into DNA, and cellular proliferation in hepatocytes transfected with pT3, miR-206 or a combination of miR-206 and sgRNAs of both *Hmgcr* and *G6pd* (sgRNAs). Cellular proliferation was evaluated via MTT. **F**–**J** The rates of ^14^C-acetate incorporation into cholesterol, levels of NADPH and R5P, the rates of ^3^H-thymidine incorporation into DNA, and cellular proliferation in hepatocytes transfected with pT3, miR-206 or a combination of miR-206 and *Hmgcr* sgRNAs. **K**–**O** Levels of R5P and NADPH, the rates of ^14^C-acetate incorporation into cholesterol, the rates of ^3^H-thymidine incorporation into DNA, and cellular proliferation in hepatocytes transfected with pT3, miR-206 or a combination of miR-206 and *G6pd* sgRNAs. Data represent mean ± SEM. **p < 0.01 and ***p < 0.001 (Fig. 6: two-way ANOVA test).

Since *Hmgcr* and *G6pd* are direct targets of miR-206, we hypothesized that miR-206 is able to disrupt the positive feedback loop between cholesterol synthesis and the PPP, thereby inhibiting DNA synthesis, cholesterol synthesis and proliferation of malignant hepatocytes. To test this, CRISPR/Cas9 technique was used to delete the binding sites of miR-206 within the 3’UTRs of both *G6pd* and *Hmgcr* in malignant hepatocytes isolated from c-Myc mice [29, 30]. Such a design disrupted the interaction of miR-206 with *G6pd* and *Hmgcr* (Supplementary Fig. 9A), allowing us to determine if *G6pd* and *Hmgcr* are required for miR-206 to inhibit cholesterol synthesis and the PPP. Overexpression of miR-206 inhibited cholesterol synthesis, the PPP and DNA synthesis, which was reflected by a significant reduction in incorporation of ^14^C-acetate into cholesterol, the PPP metabolites, ^3^H-thymidine incorporation into DNA and proliferation of hepatocytes (Fig. 6A–E). In contrast, ablating the miR-206 binding sites within the 3’UTRs of both *Hmgcr* and *G6pd* offset the inhibitory effects of miR-206 (Fig. 6A–E).

We assumed that a positive feedback loop between cholesterol synthesis and the PPP amplifies DNA synthesis and proliferation of malignant hepatocytes. Inhibition of either cholesterol synthesis or the PPP should be able to at least in part disrupt this positive feedback loop and thereby prevent DNA synthesis and proliferation. To test this, we ablated the binding sites of miR-206 within the 3’UTR of *Hmgcr* or *G6pd*. Ablation of the miR-206 binding sites within the 3’UTR of *Hmgcr* was able to recover cholesterol synthesis, the PPP, DNA synthesis and cell proliferation that were inhibited by miR-206 (Supplementary Fig. 9B, Fig. 6F–J). Similarly, ablation of the miR-206 binding sites within the 3’UTR of *G6pd* also recovered cholesterol synthesis, the PPP, DNA synthesis and proliferation (Supplementary Fig. 9C, Fig. 6K–O). These results indicate that miR-206 is able to disrupt the positive feedback loop between cholesterol synthesis and the PPP by targeting either *Hmgcr* or *G6pd*, which subsequently inhibits DNA synthesis and cell proliferation.

### MiR-206 inhibited cholesterol synthesis and the PPP in c-Myc mice

**Fig. 7:**
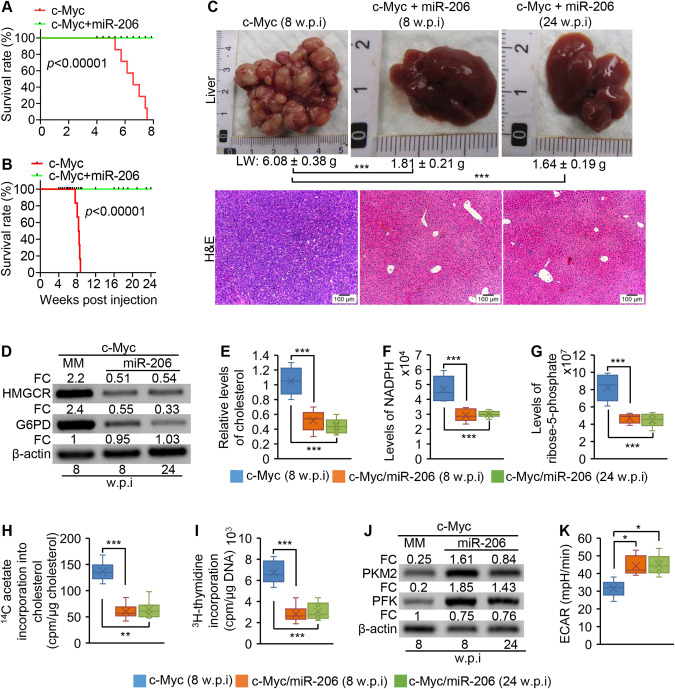
miR-206 inhibited cholesterol synthesis and the PPP but promoted glycolysis in c-Myc mice. **A** Kaplan–Meier survival curves of c-Myc/miR-206-MM (control, *n* = 6) and c-Myc/miR-206 mice (*n* = 6). Eight-week-old wild-type FVB/NJ mice were hydrodynamically injected with c-Myc and pT3-EF1α-miR-206-MM or c-Myc and pT3-EF1α-miR-206. Eight weeks post injection; mice were sacrificed for further analysis. **B** Kaplan–Meier survival curves of c-Myc/miR-206-MM (*n* = 6) and c-Myc/miR-206 mice (*n* = 6). Eight-week-old wild-type FVB/NJ mice were hydrodynamically injected with -Myc and pT3-EF1α-miR-206-MM or c-Myc and pT3-EF1α-miR-206. Twenty-four weeks post injection, mice were sacrificed for further analysis. **C** Macroscopic (upper panel) and microscopic (lower panel) appearance of livers from c-Myc/miR-206-MM (control, *n* = 6, 8 w.p.i), c-Myc/miR-206 mice (*n* = 6, 8 w.p.i) or c-Myc/miR-206 mice (*n* = 6, 24 w.p.i) stained with H&E (10X). LW: liver weight. **D** Western blot analysis of HMGCR and G6PD in pooled hepatocytes of c-Myc/miR-206-MM (*n* = 3, 8 w.p.i), c-Myc/miR-206 mice (*n* = 3, 8 w.p.i) or c-Myc/miR-206 (*n* = 3, 24 w.p.i) mouse cohort. **E** Hepatic cholesterol levels in three groups of mice. **F**, **G** Levels of NADPH and R5P in livers of three groups of mice. **H**, **I** The rates of ^14^C-acetate incorporation into cholesterol and the rates of ^3^H-thymidine incorporation into DNA in livers of three groups of mice. **J** Protein levels of PKM2 and PFK in pooled hepatocytes (*n* = 3) isolated from three groups of mice. **K** The extracellular acidification rate (ECAR) in hepatocytes isolated from three groups of mice after sequential additions of 10 mM glucose, 2 μg/mL oligomycin, and 100 mM 2-deoxyglucose. Data represent mean ± SEM. *p < 0.05; **p < 0.01; and ***p < 0.001 (Fig. 7A, B: log-rank test; Fig. 7C–K: two-way ANOVA test).

We next determined if miR-206 was able to simultaneously inhibit cholesterol synthesis and the PPP in vivo. c-Myc mice were injected with pT3-EF1α-miR-206-MM (control) or pT3-EF1α-miR-206. Eight weeks post injection, all miR-206-treated c-Myc mice were healthy, while 100% of c-Myc mice died of HCC (Fig. 7A). Upon dissection, no tumors were observed in c-Myc/miR-206 mice (Fig. 7C). The long-term survival experiment revealed that all c-Myc/miR-206 mice were healthy 24 weeks of post injection of miR-206 (Fig. 7B). Upon dissection, no tumor nodules were observed in livers of this group of c-Myc/miR-206 mice (Fig. 7C). All these findings indicated the long-term effect of miR-206 on preventing HCC. Our hypothesis is that miR-206, by disrupting the positive feedback loop between cholesterol synthesis and the PPP, inhibits HCC. As expected, miR-206 significantly reduced expression of *Hmgcr* and *G6pd* in hepatocytes of c-Myc mice (Fig. 7D). Consistent with reduced *Hmgcr* and *G6pd*, both cholesterol and the metabolites of the PPP were significantly reduced in miR-206-treated c-Myc mice (Fig. 7E–G). Incorporation of ^14^C-acetate into cholesterol and ^3^H-thymidine incorporation into DNA were reduced in miR-206-treated c-Myc mice (Fig. 7H, I). These results established that miR-206 disrupted the positive feedback loop between cholesterol synthesis and the PPP, which subsequently inhibited DNA synthesis and growth of malignant hepatocytes. Unexpectedly, miR-206 treatment significantly induced expression of genes encoding PFK (phosphofructokinase) and PKM2 (pyruvate kinase), two rate-limiting enzymes of glycolysis, in c-Myc mice (Fig. 7J). The glycolytic rate was also significantly increased in livers of c-Myc/miR-206 mice (Fig. 7K). Although miR-206 induced glycolysis, it still fully prevented c-Myc-induced HCC, further indicating that glycolysis is not required for miR-206 to inhibit HCC. This finding is consistent with our observation that ablation of glycolysis failed to prevent c-Myc-induced HCC (Fig. 3I). In sum, miR-206 disrupted the positive feedback between cholesterol synthesis and the PPP, which prevented c-Myc-induced HCC.

### HMGCR and G6PD are required for miR-206 to prevent c-Myc-induced HCC

**Fig. 8:**
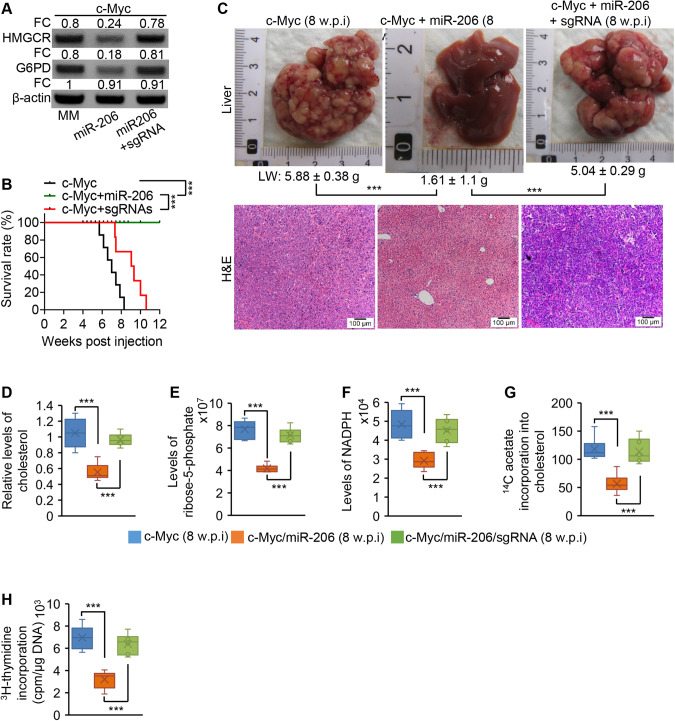
Disrupting the interaction of miR-206 with *Hmgcr* and *G6pd* impaired the ability of miR-206 to inhibit cholesterol synthesis, the PPP and HCC. **A** Protein levels of HMGCR and G6PD in pooled hepatocytes isolated from c-Myc/miR-206-MM (*n* = 3, 8 w.p.i), c-Myc/miR-206 (*n* = 3, 8 w.p.i) or c-Myc/miR-206/sgRNA (*n* = 3, 8 w.p.i). Eight week-old wild-type FVB/NJ mice were hydrodynamically injected with pT3-EF1α-cMyc and pT3-EF1α-miR-206-MM, pT3-EF1α-c-Myc and pT3-EF1α-miR-206, or a combination of pT3-EF1α-cMyc, pT3-EF1α-miR-206, and sgRNAs of *Hmgcr* and *G6pd*. **B** Kaplan–Meier survival curves of c-Myc/miR-206-MM (*n* = 6), c-Myc/miR-206 (*n* = 6) and c-Myc/miR-206/sgRNA mouse cohorts. **C** Macroscopic (upper panel) and microscopic (lower panel) appearance of livers from c-Myc/miR-206-MM (*n* = 6), c-Myc/miR-206 (*n* = 6) and c-Myc/miR-206/sgRNA mouse cohorts stained with H&E (10X). LW: liver weight. **D** Relative levels of hepatic cholesterol in three groups of mice. **E**, **F** Levels of R5P and NADPH in livers of three groups of mice. **G**, **H** The rates of ^14^C-acetate incorporation into cholesterol and the rates of ^3^H-thymidine incorporation into DNA in livers of three groups of mice. Data represent mean ± SEM. ***p < 0.001 (Fig. 8B: log-rank test; Fig. 8A, C–H: two-way ANOVA test).

We next employed an AAV8-based CRISPR/Cas9 technique to ablate the binding sites of miR-206 within the 3’UTRs of *Hmgcr* and *G6pd* in the genome of hepatocytes in c-Myc mice, which disrupted the interaction of miR-206 with *Hmgcr* and *G6pd*. Ablation of the miR-206 binding sites impaired the ability of miR-206 to inhibit expression of *Hmgcr* and *G6pd* in hepatocytes (Fig. 8A). Phenotypically, 100% c-Myc mice died of HCC within 8 weeks post injection of c-Myc (Fig. 8B). MiR-206 fully prevented c-Myc-induced HCC, while disrupting its interaction with *Hmgcr* and *G6pd* resulted in renewed growth of HCC that was fully prevented by miR-206 (Fig. 8B, C). Mechanistically, miR-206 markedly reduced hepatic cholesterol and metabolites of the PPP, while disrupting the interaction of miR-206 with *Hmgcr* and *G6pd* recovered levels of hepatic cholesterol and the metabolites of the PPP (Fig. 8D–F). As revealed by ^14^C-acetate- and ^3^H-thymidine-labeling experiments, ablating the miR-206 binding sites recovered cholesterol synthesis and DNA synthesis in miR-206-treated c-Myc mice (Fig. 8G, H). In sum, by disrupting the positive feedback loop between the cholesterol synthesis and the PPP, miR-206 inhibited growth of HCC.

## Discussion

Amplification of c-MYC has been implicated in ~27% HCC patients [31]. It has been reported that c-MYC drives cholesterol synthesis [32]; and a positive correlation between *c-MYC* and *HMGCR* was observed in HCC patients. Furthermore, we discovered that cholesterol synthesis is activated in tumors of HCC patients and high levels of *HMGCR* predicted poor survival of HCC patients. However, how cholesterol synthesis drives HCC development is poorly understood. To simulate the observation in HCC patients, we overexpressed c-Myc in livers of mice. Cholesterol synthesis is activated in livers of c-Myc mice. Mechanistically, activation of c-Myc maintained the positive feedback loop between cholesterol synthesis and the PPP. Specifically, activation of the PPP overproduced R5P and NADPH that served as substrates of DNA synthesis and the cofactor of HMGCR. Enhancement of cholesterol synthesis depleted NADPH, thereby driving the PPP. This positive feedback loop maintained rapid production of DNA and cholesterol, which are required for rapid growth and proliferation of malignant hepatocytes. These findings potentially explained the rapid development of HCC in c-Myc mice. In our previous study, we observed that miR-206 is able to suppress immunosuppression by preventing overproduction of TGFβ in malignant hepatocytes, which partially contributed to the prevention of c-Myc-induced HCC [33]. However, miR-206 was able to fully prevent c-Myc-induced HCC, while 100% of c-Myc mice died of HCC within 8 weeks post injection (Fig. 7A–C). Such a potent inhibitory effect of miR-206 on HCC led us to speculate that in addition to recovering anti-tumor immunity, other mechanism(s) are involved in miR-206-mediated inhibition of HCC in c-Myc mice. Unexpectedly, miR-206 was identified as the only miRNA that can simultaneously target both *Hmgcr* and *G6pd*. By simultaneously targeting *Hmgcr* and *G6pd*, miR-206 disrupted the positive feedback loop between cholesterol synthesis and the PPP, thereby mitigating cholesterol and DNA synthesis that are required for growth and proliferation of malignant hepatocytes. Our findings fill the knowledge gap regarding the roles of cholesterol synthesis, the PPP and glycolysis in c-Myc-driven HCC. As described above, c-Myc mouse model is an ideal model for studying the mechanisms of c-Myc-driven hepatocarcinogenesis. However, considering the heterogeneity in the functions of cholesterol synthesis, the PPP, and glycolysis in different mouse models such as transgenic AKT/Ras and β-catenin and carcinogen-induced mouse models, further study is needed for their detailed functions during hepatocarcinogenesis.

First, we proposed a new concept that cholesterol synthesis rather than cholesterol is the major contributor of HCC development. It is well-established that dysregulated cholesterol metabolism is involved in HCC development. Most of these studies consider cholesterol as a major causal factor of hepatocarcinogenesis [12, 34]. However, the major purpose of these studies is to reduce hepatic cholesterol and the findings are controversial [6, 12, 34, 35]. In fact, levels of hepatic and blood cholesterol are controlled by cholesterol synthesis and excretion and absorption of cholesterol [14]. Statins function via driving cholesterol reverse transport (RCT) rather than inhibiting hepatic cholesterol synthesis [14], which might be the major reason of these controversial findings. In this manuscript, we for the first time established that cholesterol synthesis rather cholesterol is the major contributor of hepatocarcinogenesis, which potentially provides an explanation of controversial findings on the roles of cholesterol metabolism in HCC.

Second, we observed that the PPP rather than glycolysis promoted cholesterol synthesis. Activation of the PPP and glycolysis has been observed in c-Myc-induced HCC. Cholesterol synthesis is closely connected glycolysis and the PPP and cholesterol synthesis is activated in HCC, urging us to assess if glycolysis or the PPP affects cholesterol synthesis. As described above, activation of the PPP promoted cholesterol synthesis. However, glycolysis exhibited no effect on this process. We established that cholesterol synthesis is required for c-Myc-induced HCC, which potentially explained that ablation of glycolysis did not affect growth of HCC in c-Myc mice. It is well accepted that glycolysis provides energy for rapid growth of tumors. In fact, studies of ATP production by glycolysis and oxidative phosphorylation (OXPHOS) in various types of cells and organs concluded that OXPHOS is the main contributor of ATP under aerobic conditions [16], further suggesting that glycolysis is not the major energy supply for HCC growth. This is confirmed by our observation that although miR-206 markedly promoted glycolysis (Fig. 7J, K), it fully prevented c-Myc-induced HCC (Fig. 8C). Another explanation for glycolysis to promote HCC is that the pathway provides important biosynthetic precursors [36]. In fact, the majority of the biomass of proliferating cells is derived from amino acids rather than glucose [36]. The PPP supports cancer cell growth by providing NADPH for cholesterol synthesis and generating R5P for DNA synthesis [37]. It is the PPP rather than glycolysis is a major contributor of c-Myc-induced HCC.

The third novel observation is the positive feedback loop between cholesterol synthesis and the PPP in c-Myc-induced HCC. Cholesterol accumulation and activation of the PPP have been observed in HCC patients [6, 38]. Cholesterol accumulation in the liver contributes to lipotoxicity, hepatic inflammation, and fibrosis, which have been considered key causal factors of HCC [34]. Conversely, some clinical studies have revealed that high levels of cholesterol are, in fact, associated with a reduced risk of HCC [34]. Our findings address the controversy in that cholesterol synthesis rather than cholesterol itself is a major driver of hepatocarcinogenesis. In detail, enhancement of cholesterol synthesis, by depleting NADPH, drives the PPP, which produces R5P for DNA synthesis and NADPH for cholesterol synthesis. It is our speculation that rapid growth of cancer cells depletes cholesterol and R5P, which further enhances the positive feedback loop. Indeed, dietary cholesterol treatment significantly reduced enzyme activity of G6PD [39], further confirming our findings.

Somewhat unexpectedly, miR-206 was identified as the only miRNA that can target both *HMGCR* or *G6PD* by interacting with two miR-206 binding sites within the 3’UTR of *HMGCR* or *G6PD*. Consistently, levels of *HMGCR* and *G6PD* are significantly increased in tumors of HCC patients; and a positive association was observed between *HMGCR* and *G6PD* in HCC (Supplementary Fig. 10). More importantly, high levels of their expression predicted poor survival among HCC patients. This unexpected finding indicated the strong inhibition of miR-206 on cholesterol synthesis and the PPP. Considering the potent inhibitory effects of miR-206 on HCC, we speculated that miR-206 fully prevents HCC growth via multiple mechanisms. In addition to restoring antitumor immunity [33], miR-206-mediated inhibition of cholesterol synthesis and the PPP at least in part contributes to HCC prevention. Our final goal is to develop miR-206 as a therapeutic drug for the treatment of HCC. The novel findings in this study provide additional layer of evidence that miR-206 prevents growth of HCC by cutting off building blocks of hepatocyte proliferation.

## Materials and methods

### Establishment of c-Myc HCC mice

Eight-week-old wild-type male FVB/N mice maintained on normal diet were hydrodynamically injected with either 5 μg pT3-EF1α-cMyc and 0.2 μg pCMV/*SB* (*n* = 6) or 5 μg pT3-EF1α and 0.2 μg pCMV/*SB* (control, *n* = 6), as described previously [20]. Eight weeks post injection, mice were sacrificed for further analysis. Mice were randomly allocated to experimental group. The treatment assignment and the group allocation was completely blinded to investigators. Mice were housed, fed, and monitored in accordance with protocols approved by the committee for animal research at the Hubei University of Chinese Medicine and the University of Minnesota.

### Metabolomics by UPLC-MS/MS

50 mg of livers (in liquid nitrogen) were thawed in a 2 mL EP tube on ice. After 500 uL of pre-cooled extractant (70% methanol aqueous solution) and small steel balls were added to the EP tube, liver tissues were homogenized at 30 Hz for 30 s for four times. Homogenized livers were shaken at 1500 r/min for 5 min, incubated for15 min on ice, and centrifuged with 12,000 r/min at 4 °C for 10 min. The supernatant was collected for UPLC-MS/MS analysis using an LC-ESI-MS/MS system (UPLC, Shim-pack UFLC SHIMADZU CBM30A system; MS, AB SCIE QTRAP System). The detailed procedure of UPLC analysis is included in Supplementary Materials and Methods.

### Cholesterol synthesis assay via incorporation of ^14^C-acetate sodium

The rate of cholesterol synthesis is high in the morning hours [40]. Therefore, mice were sacrificed between 10 am and 12 pm to collect liver samples. Liver homogenates were prepared based on the protocol described previously [41]. Cold liver homogenates (400 mg) were incubated in the presence of 2 mM sodium acetate containing 16.7 µCi of ^14^C sodium acetate (PerkinElmer), as reported [42]. After incubation, the liver homogenates were transferred to new 20 mL glass tubes with cap and further saponified for three hours at 70 °C in the presence of 5 mL ethanol and 0.5 mL 90% potassium hydroxide (KOH). Cholesterol was precipitated as digitonide and its radioactivity determined in a liquid scintillation counter.

### Effects of NAD and NADP on cholesterol synthesis

Liver homogenates were prepared as described previously [24]. After centrifuge at 800 × *g* for 10 min at −1 °C, the supernatant was collected and supplemented with glucose-6-phosphate (G6P, 20 ×10^–3 ^M), nicotinamide adenine dinucleotide (NAD, 0.8 ×10^–3 ^M) or nicotinamide adenine dinucleotide phosphate (NADP, 0.8 ×10^–3 ^M), potassium acetate (2 ×10^–3 ^M) and 16.7 µCi of ^14^C sodium acetate. After one hour of incubation at 37 °C, the liver homogenate was saponified by adding 90% KOH. Cholesterol was precipitated as digitonide and its radioactivity was determined.

### Incorporation of ^3^H-thymidine into DNA

Primary hepatocytes were plated in collagen-coated 35 mm dishes containing DMEM medium. Forty-eight hours after transfection of pT3-EF1α (control), pT3-EF1α-cMyc or a combination of pT3-EF1α-cMyc and pT3-EF1α-shG6pd or pT3-EF1α-shHmgcr, the medium was replaced with fresh media containing 7.5 µCi ^3^H-thymidine. After 24 h, cells were harvested to analyze incorporation of ^3^H-thymidine DNA by standard methods [43, 44].

### In Vitro analysis of ^14^C-acetate incorporation into cholesterol

After 4 h of incubation with 10 μCi [2–^14^C] acetate, murine hepatocytes were washed with cold PBS twice, solubilized with 0.1 M sodium hydroxide, and saponified. Nonsaponifiable lipid was extracted with isohexane. Labeled cholesterol was measured by flow scintillography after HPLC.

### Expression vectors of miR-206 and c-Myc

A mouse DNA fragment containing miR-206 precursor was inserted into pT3-EF1α vector, referred to as pT3-EF1α-miR-206 [20]. To rule out a non-specific effect of the vector, we generated a miR-206 mis-matched-expression vector by mutating the seed region of miR-206 (pT3-EF1α-miR-206-MM). pCMV/*Sleeping Beauty* transposase (pCMV/SB) and pT3-EF1α-c-Myc have been described previously [20].

### CRISPR/Cas9 to ablate miR-206 binding sites within 3’UTRs of *Hmgcr* and *G6pd*

The locations of sgRNA pairs were selected at the boundary of the miR-206 binding site within the 3’UTRs of *Hmgcr* and *G6pd*. Two sgRNAs for each of the miR-206 binding sites were designed by CRISPR and synthesized in IDT (Coralville, IA) [45]. Four pairs of sgRNAs were further cloned into pX601-AAV8-CMV-SaCas9 (Addgene, Watertown, MA), termed AAV8-sgRNA. The viruses of AAV8-SaCas9 and AAV8-sgRNA were packaged and tittered in the Viral Vector and Cloning Core at the University of Minnesota. To delete the miR-206 binding sites, Group I mice (*n* = 6) received 5 μg pT3-EF1α-c-Myc, 10 μg pT3-EF1α-miR-206-MM and 0.6 μg pCMV/SB; Group II mice (*n* = 6) received 5 μg pT3-EF1α-c-Myc, 10 μg pT3-EF1α-miR-206, and 0.6 μg pCMV/SB, and 5 ×10^11^ GC AAV8-SaCas9 viruses; and Group III mice (*n* = 6) received 5 μg pT3-EF1α-c-Myc, 10 μg pT3-EF1α-miR-206, 0.6 μg pCMV/SB, and 5 ×10^11^ GC AAV8-sgRNA viruses. Eight weeks post-injection, mice were sacrificed for further analysis.

### Statistical analysis

Data were analyzed for normal distribution with a Shapiro–Wilk normality test using Prism Software® (GraphPad, San Diego, CA). Data represent mean ± standard error of the mean (SEM). Sample size was determined by power analysis to provide sufficient statistical power to detect differences. For the normally distributed data with equal variance, two-tailed Student’s *t* test was used to compare two datasets and two-way ANOVA (analysis of variance) analysis was performed to compare three or more datasets. All experiments were performed in triplicate and repeated at least three times. Survival rates were evaluated via Kaplan–Meier analysis and compared using log-rank test. *P* < 0.05 was considered statistically significant.

### Supplementary information

Supplementary materials

## Data Availability

All data generated or analyzed during this study are included in the manuscript and the Supplementary Materials. Materials used in this study are available upon reasonable request.
