# Supplementary material for: A positive feedback between cholesterol synthesis and the pentose phosphate pathway rather than glycolysis promotes hepatocellular carcinoma
Source: Oncogene. 2023 Jun 26;42(39):2892–904. doi: 10.1038/s41388-023-02757-9 (PMC10516751; doi:10.1038/s41388-023-02757-9)
Supplement: Supplementary file 1 — Supplementary materials [file 41388_2023_2757_MOESM1_ESM.docx]

**Supplementary Materials and Methods**

**UPLC analysis**

The UPLC analyses were performed based on the following conditions: 5 µm SeQuant ZIC-pHILIC column (2.1 × 100 mm), 40 °C, 0.4 mL/min of flow rate, 2 μL of injection volume, solvent system: water with 10 mmol/L ammonium acetate and 0.3% ammonia (A) and 90% acetonitrile water (B), and gradient program of 5:95 (A/B,V/V) at 0 min, 50:50 at 9.5 min, 5:95 at 11.1 min, and 5:95 at 14.0 min. The MS conditions with ESI source operation parameters were as follows: source temperature 450 °C, ion spray voltage (IS) 5500 V (positive), -4500 V (negative), ion source gas I (GSI), gas II (GSII), curtain gas (CUR) at 40, 55, and 35.0 psi respectively, and the medium collision gas (CAD). Instrument tuning and mass calibration were performed with 10 and 100 μmol/L polypropylene glycol solutions in QQQ and LIT modes, respectively. A specific set of MRM transitions were monitored for each period according to the metabolites eluted within this period.

**Identification of miRNAs that target *HMGCR* and *G6PD***

To identify miRNAs that can target *HMGCR* and *G6PD*, we downloaded the murine and human Ago HITS-CLIP databases of *HMGCR* and *G6PD* (high-throughput sequencing of RNAs isolated by crosslinking immunoprecipitation from Argonaute protein complex).[^1^](#_ENREF_1) Database comparison between human and mouse identified 44 miRNAs that potentially bind to the 3’UTR of *HMGCR* and *G6PD*. To exclude the false positive peaks of Ago-HITs-CLIP, we further used DIANA-microT-CDS to scan the 3’UTRs of murine and human *HMGCR* and *G6PD*. By doing so, we identified miR-206 as the only miRNA that have two binding sites within the 3’UTR of both mouse and human *HMGCR* and *G6PD*.[^2^](#_ENREF_2) In addition, prediction from *in silico* algorithms showed that 3' UTRs of both human and mouse *HMGCR* and *G6PD* mRNAs are 100% complementary to the miR-206 5' seed region, exhibiting the highest prediction scores and binding energy. These findings led us to focus on miR-206.

**Survival Analysis of HCC Patients**

The association between levels of *HMGCR* and *G6PD* and survival of HCC patients was determined via Kaplan–Meier analysis of LIHC patients in the TCGA database. *P* ≤ 0.05 was considered as the statistical significance.

**Correlation Analyses**

The correlation analysis was calculated using the Spearman correlation coefficients, which was used to estimate 2-tailed *P* values of the correlations.

**Hepatocyte Isolation**

Mouse livers were enzymatically digested, and hepatocytes were isolated by low speed centrifugation. Briefly, dissected livers were washed with cold HBSS and teased into pieces with forceps, and then incubated with Digestion Buffer (10 mL Collagenase Ⅳ (1 mg/mL) and 5 µL DNase Ⅰ (100 U/µL)) at 37 °C for 30 minutes. The cells were poured through a 70 μm sterile nylon filter and centrifuged at 50 x g for 5 minutes at 4 °C to collect hepatocytes.

**Cell Transfection**

Hepa1-6 (ATCC, Cat. No. CRL-1830™) were purchased from ATCC. The Mycoplasma PCR Detection Kit (Sigma, USA) was routinely employed to exclude mycoplasma contamination. STR profiling was performed for authenticating Hepa1-6 cells. 5×10^4^ of Hepa1-6 or murine primary hepatocytes were seeded onto a 24-well plate and allowed to adhere overnight. Cells were transfected with 500 ng/well of pT3-EF1α-miR-206, pT3-EF1α-c-Myc or pT3-EF1α-miR-206-MM plasmids using Lipofectamine 3000 Transfection Reagent (Invitrogen). Forty-eight hours post transfection, cells were washed with cold PBS and collected for further analysis.

**HMGCR Enzyme Activity Assay**

HMGCR enzyme activity in the liver was determined using HMG-CoA Reductase Activity Assay Kit (Colorimetric) according to the manufacturer’s instructions (AbCam, Cat. No. ab204701).

**Reporter Vector Construction and Luciferase Assay**

To generate luciferase constructs, the 3' UTRs of *HMGCR* and *G6PD* were amplified from mouse and human cDNA using PCR and inserted into the pMiR-Reporter vector (Ambion, Agawam, CA), referred to, respectively, as pMiR-Hmgcr, pMiR-HMGCR, pMiR-G6pd and pMiR-G6PD. Two bases of the binding sites for miR-206 within the 3’UTR of *Hmgcr* or *G6pd* were mutated using QuikChange II Site-Directed Mutagenesis Kit (Agilent Technologies, Santa Clara, CA) per the manufacturer’s instructions, and referred to as pMiR-Hmgcr-Mu, pMiR-G6pd-Mu, pMiR-HMGCR-Mu and pMiR-G6PD-Mu. Luciferase Assays were performed following the protocol of Dual-Luciferase Reporter Assay System (Promega, Madison, WI).

**RNA Isolation and Quantitative Reverse Transcription-PCR (qRT-PCR).** Total RNA was isolated with the Monarch Total RNA Miniprep Kit (Biolabs, Boston, MA). Briefly, 1 μg RNA was used for cDNA synthesis with Superscript III reverse transcription reagent (Invitrogen, Carlsbad, CA) to assess gene expression; and 50 ng RNA was used for cDNA synthesis with the TaqMan MicroRNA Reverse Transcription Kit (Applied Biosystems) to detect miRNA expression. PCR amplification was performed as previously described.[^3^](#_ENREF_3) Primers for qRT-PCR were designed with Primer Express software (Applied Biosystems); and relative changes in gene and miRNA expression were determined using the 2^-ΔΔCt^ method.

**Western Blots and Antibodies**

Proteins were extracted from cells and tissues using RIPA buffer (Cell Signaling Technology, Beverly, MA) in combination with proteases and phosphatase inhibitors (Roche, Indianapolis, IN, USA). Protein concentrations were measured using Pierce BCA Protein Assay Kit and 25~50 μg of total lysate was loaded and immunoblotted. Antibodies to c-Myc (Cat# ab32072) and G6PD (Cat# ab231828) were purchased from Abcam. Antibody to HMGCR (Cat# MA5-32521) was purchased from ThermoFisher. Anti-β-actin antibody (NB600-501) was obtained from Novus Biologicals.

**Measurement of Hepatic Cholesterol**

Levels of hepatic cholesterol were determined according to the manufacturer’s instructions (MAK043, Sigma).

**Glycolysis Assay**

Primary hepatocytes were incubated with glucose-free Seahorse XF base medium without phenol red and supplemented with 2 mM glutamine, 1 mM pyruvate, and 5 mM HEPES (pH 7.4) at 37 °C in a non-CO_2_ chamber for 1 hour prior to glycolytic rate assay (Cat# 103344-100; Seahorse XF glycolytic rate assay kit; Agilent Technologies, Billerica, MA). Quantitative real-time measurements of glycolytic rate and glycolytic capacity (extracellular acidification rate [ECAR]) were assessed for hepatocytes using a Seahorse XFe96 Analyzer according to manufacturer's recommendations.

**Supplementary Table 1 Two binding sites of miR-206 within the 3’UTRs of both human and murine *HMGCR* and *G6PD*.**

| **miRNAname** | **Gene**  **Name** | **narrowStart** | **narrowEnd** | **broadStart** | **broadEnd** | **clipExp**  **Num** | **RBP** | **microT** |
| --- | --- | --- | --- | --- | --- | --- | --- | --- |
| hsa-miR-206 | HMGCR | 74654606 | 74654634 | 74654606 | 74654634 | 5 | AGO1-4 | 1 |
| hsa-miR-206 | HMGCR | 74657621 | 74657627 | 74657600 | 74657628 | 3 | AGO1-4 | 1 |
| mmu-miR-206 | Hmgcr | 96649266 | 96649272 | 96649265 | 96649293 | 4 | Ago1-4,Ago2 | 1 |
| mmu-miR-206 | Hmgcr | 96652676 | 96652704 | 96652676 | 96652704 | 3 | Ago2 | 1 |
| hsa-miR-206 | G6PD | 153759776 | 153759782 | 153759775 | 153759803 | 4 | AGO1-4,AGO2 | 1 |
| hsa-miR-206 | G6PD | 153760044 | 153760050 | 153760043 | 153760071 | 6 | AGO1-4,AGO2 | 1 |
| hsa-miR-206 | G6PD | 153760112 | 153760118 | 153760111 | 153760139 | 9 | AGO1-4,AGO2 | 1 |
| mmu-miR-206 | G6pd | 74409673 | 74409694 | 74409673 | 74409701 | 1 | Ago2 | 1 |
| mmu-miR-206 | G6pd | 74409991 | 74409997 | 74409990 | 74410018 | 2 | Ago2 | 1 |

**
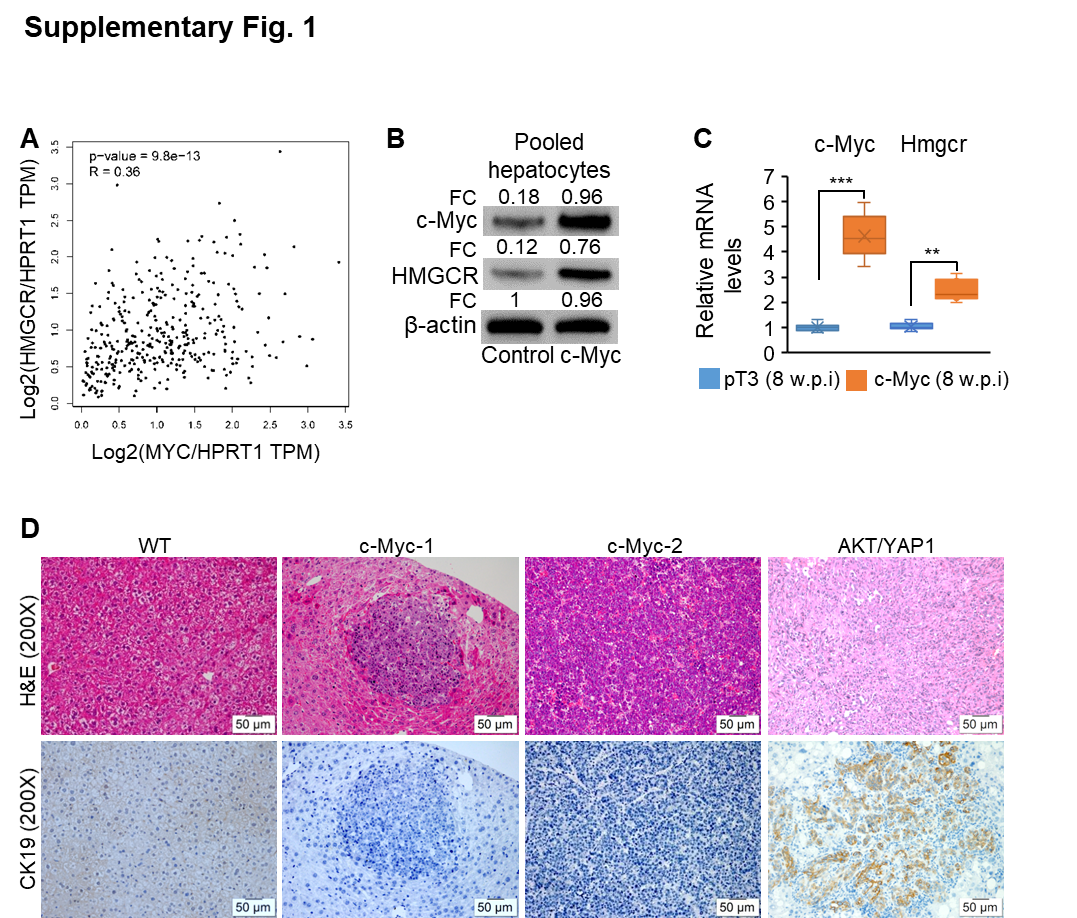
**

**Supplementary Fig. 1 The positive correlation between *c-MYC* and *HMGCR* in tumors of HCC patients. (A)** The positive correlation between *c-MYC* and *HMGCR* in tumors of HCC patients from the TCGA database. HPRT1 served as an internal control. Spearman correlation was used to evaluate the correlation between *c-MYC* and *HMGCR*. (**B**) Protein levels of c-MYC and HMGCR in pooled hepatocytes isolated from pT3 (*n*=3) and c-Myc (*n*=3) mouse cohorts. (**C**) mRNA levels of *c-Myc* and *Hmgcr* in hepatocytes isolated from pT3 (*n*=6) and c-Myc (*n*=6) mouse cohorts. (**D**) Staining of H&E or CK19 (ICC marker) of livers from mice hydrodynamically injected with pT3-EF1α (pT3, *n*=6), pT3-EF1α-c-Myc (*n*=6), or a combination of pT3-EF1α-AKT and pT3-EF1α-YAP (AKT/YAP, *n*=6). Data represent mean ± SEM. ***p* < 0.01 and ****p* < 0.001 (Fig. 1A: Spearman correlation; Fig. 1B-C: two-tailed student’s *t* test)


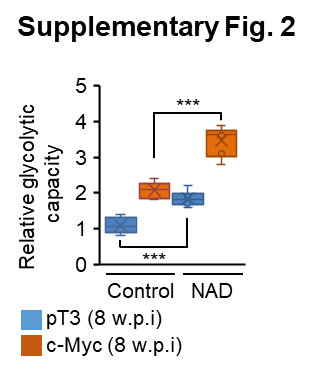


**Supplementary Fig. 2 NAD drove glycolysis in the liver. (A)** Relative levels of glycolytic capacity in livers of pT3 (*n*=6) and c-Myc (*n*=6) mouse cohorts treated with NAD. Data represent mean ± SEM. ****p* < 0.001 (Fig. 2: two-way ANOVA test)

**
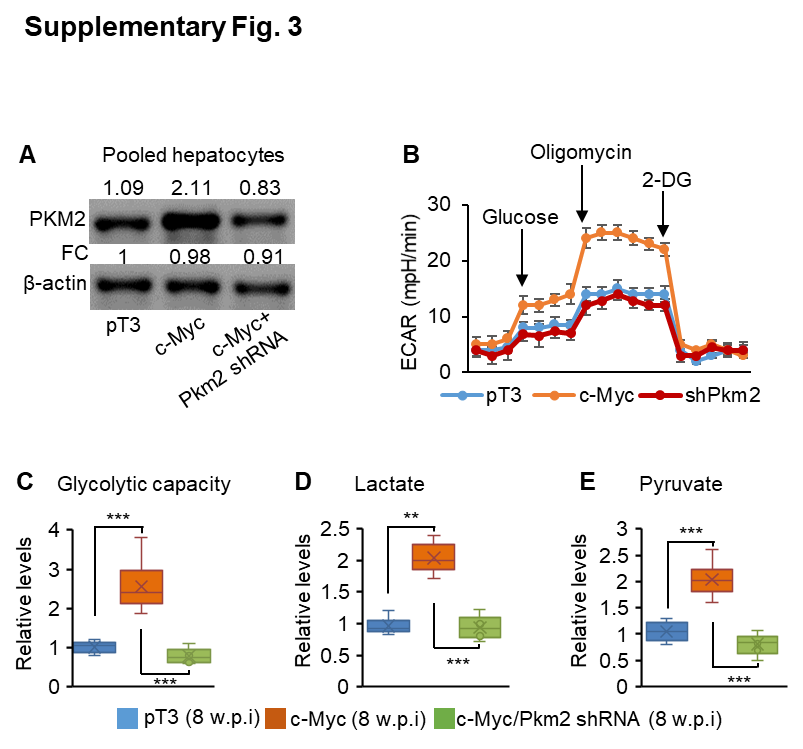
**

**Supplementary Fig. 3 Knockdown of *Pkm2* impaired glycolysis in hepatocytes of c-Myc mice. (A)** Protein levels of PKM2 in pooled hepatocytes isolated from pT3 (*n*=3), c-Myc (*n*=3) or c-Myc/Pkm2 shRNA (*n*=3) mouse cohort. **(B-C)** Glycolytic capacity in hepatocytes isolated from mice injected with pT3 (*n*=6), c-Myc (*n*=6) or c-Myc/Pkm2 shRNA (*n*=6). (**D-E**) Relative levels of acetate and pyruvate in hepatocytes isolated from three groups of mice. Data represent mean ± SEM. ****p* < 0.001 (Fig. 3: two-way ANOVA test)

**
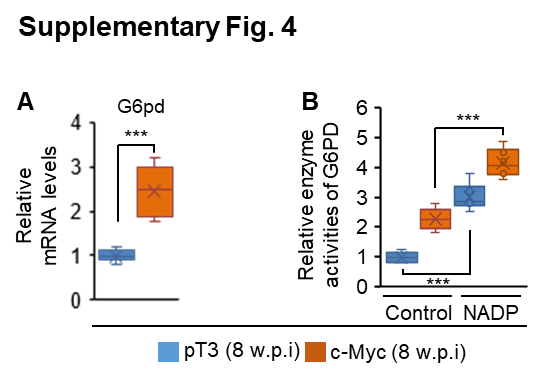
**

**Supplementary Fig. 4 NADP promoted the PPP in the liver. (A)** Relative levels of *G6pd* mRNA in livers of pT3 (*n*=6) and c-Myc (*n*=6) mouse cohorts. (**B**) Relative enzyme activity of G6PD in livers of pT3 (*n*=6) and c-Myc (*n*=6) mouse cohorts treated with NADP. Data represent mean ± SEM. ****p* < 0.001 (Fig. 4A: two-tailed student’s *t* test; Fig. 4B: two-way ANOVA test)

**
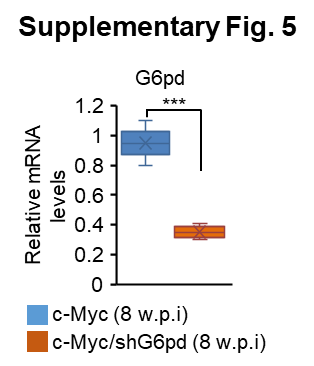
**

**Supplementary Fig. 5** Relative levels of *G6pd* mRNA in livers of c-Myc mice treated with scramble (*n*=6) or *G6pd* shRNA (*n*=6) mouse cohort. Data represent mean ± SEM. ****p* < 0.001 (Fig. 5: two-tailed student’s *t* test)


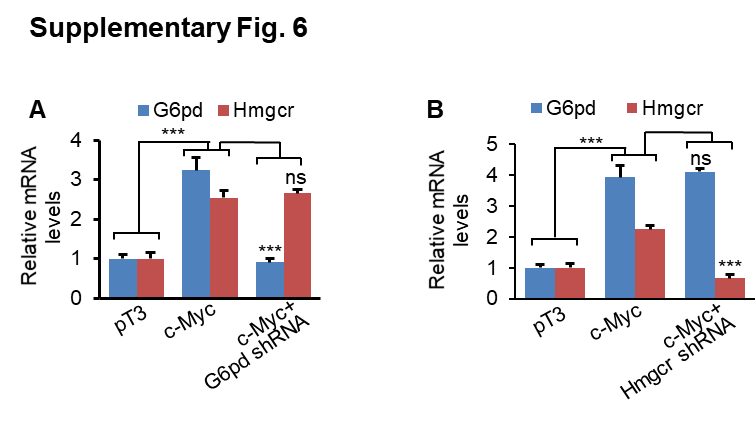
**Supplementary Fig. 6 mRNA levels of *Hmgcr* and *G6pd* in hepatocytes treated with pT3, c-Myc or a combination of c-Myc and *Hmgcr* shRNA or *G6pd* shRNA.** Data represent mean ± SEM. ****p* < 0.001 and ns: no significance (Fig. 6: two-way ANOVA test)


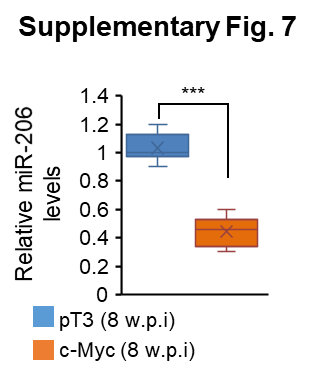


**Supplementary Fig. 7 Levels of miR-206 in livers of pT3 (*n*=6) and c-Myc mice (*n*=6).**  Data represent mean ± SEM. ****p* < 0.001 and ns: no significance (Fig. 7: two-tailed student’s *t* test)


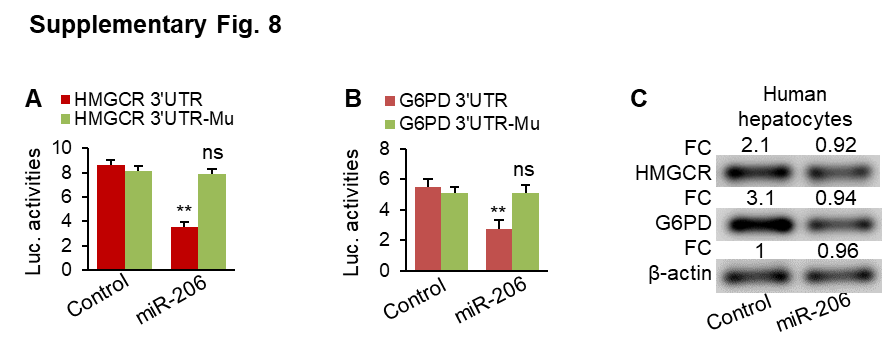


**Supplementary Fig. 8 miR-206 inhibited expression of *HMGCR* and *G6PD* in human hepatocytes by binding to their 3’UTRs. (A-B)** miR-206 markedly reduced luciferase activity of the reporter construct containing wild-type 3’UTRs of human *HMGCR* and *G6PD*. Mutation of two miR-206 binding sites within the 3’UTRs of *HMGCR* and *G6PD* nullified the ability of miR-206 to inhibit luciferase activity. (**C**) Protein levels of HMGCR and G6PD in human hepatocytes transfected with pT3-EF1α-miR-206-MM (control) or pT3-EF1α-miR-206. Data represent mean ± SEM. ***p* < 0.01 and ns: no significance (Fig. 8A-B: two-tailed student’s *t* test)


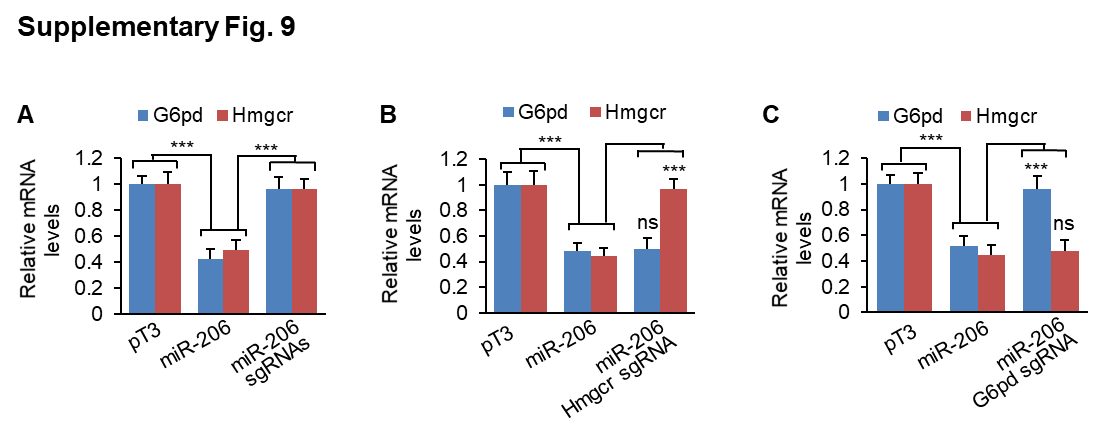
**Supplementary Fig. 9 Ablation of the miR-206 binding sites within the 3’UTRs of *Hmgcr* and *G6pd* impaired the ability of miR-206 to inhibit expression of *Hmgcr* and *G6pd*. (A)** mRNA levels of *Hmgcr* and *G6pd* in murine primary hepatocytes transfected with pT3-EF1α-miR-206-MM (control), pT3-EF1α-miR-206 or a combination of pT3-EF1α-miR-206 or sgRNAs of both *Hmgcr* and *G6pd*. (**B**) mRNA levels of *Hmgcr* and *G6pd* in murine primary hepatocytes transfected with pT3-EF1α-miR-206-MM (control), pT3-EF1α-miR-206 or a combination of pT3-EF1α-miR-206 and sgRNAs of *Hmgcr*. (**C**) mRNA levels of *Hmgcr* and *G6pd* in murine primary hepatocytes transfected with pT3-EF1α-miR-206-MM (control), pT3-EF1α-miR-206 or a combination of pT3-EF1α-miR-206 and sgRNAs of *G6pd.* ****p* < 0.001 and ns: no significance (Fig. 9: two-way ANOVA test)

**
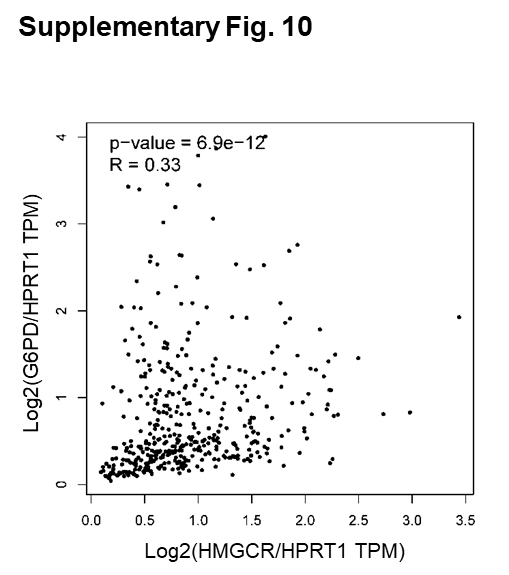
**

**Supplementary Fig. 10 The positive correlation between *HMGCR* and *G6PD* in tumors of HCC patients from TCGA database.** HPRT1 served as an internal control. Spearman correlation was used to evaluate the correlation between *G6PD* and *HMGCR*.

**References**

1. Li J-H, Liu S, Zhou H, et al. starBase v2. 0: decoding miRNA-ceRNA, miRNA-ncRNA and protein–RNA interaction networks from large-scale CLIP-Seq data. Nucleic Acids Research 2014;42:D92-D97.

2. Paraskevopoulou MD, Georgakilas G, Kostoulas N, et al. DIANA-microT web server v5. 0: service integration into miRNA functional analysis workflows. Nucleic Acids Research 2013;41:W169-W173.

3. Wu H, Tao J, Li X, et al. MicroRNA-206 prevents the pathogenesis of hepatocellular carcinoma by modulating expression of met proto-oncogene and cyclin-dependent kinase 6 in mice. Hepatology 2017;66:1952-1967.
